# Maternal Intake of Probiotics to Program Offspring Health

**DOI:** 10.1007/s13668-022-00429-w

**Published:** 2022-08-20

**Authors:** Céline Cuinat, Sara E. Stinson, Wendy E. Ward, Elena M. Comelli

**Affiliations:** 1 Department of Nutritional Sciences, Faculty of Medicine, University of Toronto, Toronto, ON Canada; 2 Department of Kinesiology, Faculty of Applied Health Sciences, Brock University, St. Catharines, ON Canada; 3 Joannah and Brian Lawson Centre for Child Nutrition, Faculty of Medicine, University of Toronto, Toronto, ON Canada

**Keywords:** Developmental Origins of Health and Disease, Probiotics, Nutritional programming, Gut microbiota, Pregnancy, Infant, Sex, Epigenetics, Human, Mouse, Rabbit, Pig, Rat

## Abstract

***Purpose of Review*:**

Probiotics intake may be considered beneficial by prospective and pregnant mothers, but their effects on offspring development are incompletely understood. The purpose of this review was to examine recent pre-clinical and clinical studies to understand how maternal probiotics exposure affects offspring health outcomes.

***Recent Findings*:**

Effects were investigated in the context of supporting offspring growth, intestinal health, and gut microbiota, preventing allergic diseases, supporting neurodevelopment, and preventing metabolic disorders in pre-clinical and clinical studies. Most human studies focused on infancy outcomes, whereas pre-clinical studies also examined outcomes at adolescence and young adulthood. While still understudied, both pre-clinical and clinical studies propose epigenetic modifications as an underlying mechanism. Optimal timing of intervention remains unclear.

***Summary*:**

Administration of selected probiotics to mothers has programming potential for sustaining life-long health of offspring. Administration protocols, specific windows of susceptibility, and individual-specific responses need to be further studied.

## Introduction

Probiotics are defined as “live microorganisms that, when administered in adequate amounts, confer a health benefit on the host” [1]. Probiotics can be delivered in various modalities such as dietary supplements, food products, or drugs, targeting individuals across the entire life spectrum. Probiotics stem from various taxa including both prokaryotic and eukaryotic microorganisms and display common and/or strain-specific benefits such as regulation of intestinal transit, competitive exclusion of pathogens, vitamin and specific bioactive synthesis, intestinal barrier enhancement, or immunomodulation [1]. The gut ecosystem is a determinant of health and a main target of probiotics administration. Established in early life, altered dynamics in this process are associated with disease later in life, such as asthma [2], allergy [3], eczema [3], obesity [4, 5], and susceptibility to infection [6]. Several probiotics have been studied for their role in early stages of life and ability to sustain health in mothers and children. Substantiated benefits in the context of eczema for lactobacilli alone or in combination with *Bifidobacterium* species administered to mothers or infants [7] have been translated into guidelines by the World Allergy Organization in 2015 [8]. These guidelines indicate that probiotic consumption during pregnancy might be beneficial for pregnant or breastfeeding women at high risk for having a child who develops allergies. Additional benefits of maternally administered probiotics include improvement of metabolic parameters (insulin levels and insulin resistance, very low density lipoprotein and total cholesterol concentration) in gestational diabetes mellitus (GDM) [9], reduced rectal and vaginal Group B Streptococci colonization before parturition (important to prevent offspring mortality caused by Early Onset Group B *Streptococcus* disease) [10], and reduced incidence of mastitis [11]. These studies are sparse and heterogeneous in terms of probiotics usage, vehicle, and dosage. Because maternal exposures such as metabolic syndrome and infection are associated with offspring outcomes [12, 13], it is likely that offspring can also be affected by maternal use of probiotics. In fact, maternal probiotics administration was shown to result in temporary colonization of the offspring and/or modulation of the offspring microbiota [14, 15].

In a recent meta-analysis, probiotic consumption during pregnancy and/or lactation was shown to be generally safe for pregnant mothers in terms of gastrointestinal symptoms, tachycardia, vaginal discharge, eczema, and headache [16]. Only one probiotic mix containing *Lacticaseibacillus rhamnosus* GR-1 and *Limosilactobacillus reuteri* RC-14 was associated with an increased risk of vaginal discharge and changes in stool consistency after consumption during the first and second trimesters of pregnancy [17]. Direct probiotic administration to infants between birth and 2 years of age has also been reported as safe. This was shown through a systematic review that stratified the incidence of an adverse effect according to the infant’s health condition (healthy, low-birth weight, dermatitis, diarrhea, or formula-fed) [18]. The utilization of probiotics during these sensitive periods of life could therefore be considered to support infant health. A survey conducted in Canada suggests that the public already utilizes probiotics as 50.8% of women with a child aged 2 years or younger reported giving a probiotic product to their infant. Responses came from 413 mothers enrolled in the Alberta Pregnancy Outcomes and Nutrition (APrON) study in 2012 [19].

It is known that an altered maternal microbiota during pregnancy can affect offspring microbiota establishment, immune development, and metabolic health throughout life [20–22]. Thus, interventions targeting the maternal microbiota, such as probiotics, may have the potential to program offspring health. The purpose of this review was to examine recent literature from pre-clinical and clinical studies on the effects of probiotic administration started during the pre-conception period or pregnancy on offspring outcomes.

## Search Strategy and Selection Criteria

PubMed, Embase, and Cochrane Library were searched for original research articles including pre-clinical (animal) and clinical studies and meta-analyses over the last 5 years (January 2017–March 2022). The search terms used were as follows: “probiotics”, “gestation”, “pregnancy”, “lactation”, “pre-conception” or “preconception”, “pre-mating” or “premating”, “pre-conceptional”, combined or not with “offspring”, “infant”, “baby” and complemented with manual inspection of reference lists of the selected articles. Articles were selected if they included a probiotic intervention starting at pre-conception and/or during pregnancy and assessed offspring outcomes. Studies of probiotics administered in combination with other ingredients were included. We included studies using substantiated probiotics strains, according to the Food and Agriculture Organization of the United Nations (FAO) guidelines [23], as well as strains for which research is ongoing, especially at the clinical stage.

Articles were screened to retrieve information about the probiotic strains, dose, vehicle, and supplementation period and this information is reported in tabulated form according to the Population, Interventions, Comparisons, Outcomes and Study designs (PICOS) elements [24] and grouped as pre-clinical (Table 1) or clinical (Table 2) studies. Findings are discussed according to 6 offspring outcomes identified across the studies: (1) growth and anthropometric indices at birth (19 articles); (2) intestinal barrier and gut health (14 articles); (3) neurodevelopment and anxiety-like behavior (8 articles); (4) allergic diseases (12 articles); (5) metabolic disorders (14 articles); and (6) intestinal microbiota (18 articles).

**Table 1:** Pre-clinical studies evaluating probiotic administration starting before or during gestation on offspring outcomes

Condition, animal model		Probiotic, dose, vehicle, time	Offspring outcomes (compared to control)	Effect on gut microbiota in dams and offspring	Ref
*Before pregnancy*					
**Metabolic disorders**					
	SPF C57BL/6 J mice receiving a HFD or not since preconception ± probiotics **Dams** *n* = 9–10/group **Offspring** *n* = 27–30/group (male and female)	*Bifidobacterium breve* DM8310, *Lactobacillus acidophilus* DM8302, *Lacticaseibacillus casei* DM8121, *Streptococcus thermophilus* DM8309 2 × 10^9^ CFU/day by gavage 6 weeks before mating–PND21	↓ body weight until PND42 PND21: ↓ total cholesterol, LDL Male: ↓ HDL PND42: ↓ total cholesterol, LDL Female: ↓ glucose and insulin. ↑ HDL compared to control diet	**Dams** ↓ Bacteroidetes, ↑ *Bacteroidetes S24-7*, *Allobaculum*, *Sutterella* **Offspring** PND21: Ameliorate HFD-induced dysbiosis (*Prevotella*, *Bacteroides*, *Bacteroidales* S24-7). Female: ↑ ⍺-diversity PND42: No effect on β-diversity. Female: Ameliorate dysbiosis (↑ *Bacteroidaceae*, *Lachnospiraceae*, *Sutterella*; ↓ *Lactobacillus*, *Prevotella*, *Helicobacter*, *Rikenellaceae*, *Parabacteroides*)	[93]
**Parasite infection**					
	Swiss mice ± probiotics, inoculated with *Toxocara canis* on GD14 **Dams** *n* = 8/group **Offspring** n = 62–65/group (male and female)	*Saccharomyces boulardii* CNCM I-745 1 × 10^7^ CFU/day, added in feed 2 weeks before mating–PND21	PND21: ↓ 42% in the number of larvae transmitted, ↓ 50% larvae found in the brain	Not assessed	[110]
*During pregnancy*					
	Wistar rats ± probiotics **Dams** n = 6/group **Offspring** n = 48/group (male and female)	*Limosilactobacillus fermentum* CECT5716 1 × 10^10^ CFU/day by gavage GD1–PND14	No effect on body weight or BMI PND14: Intestine: ↑ IgA Plasma: ↑ IgG2a, ↓ IgG2c, ↑ Th2-type Ig, no change cytokines; ↓ in palmitic acid and total saturated fatty acids, ↑ plasma linoleic acid, eicosadienoic acid, 17:0 and 22:0 carbons fatty acids	**Dams & Offspring** PND14: No effect on cecal microbiota composition, diversity, or richness PND21: Strain detected in all the dams cecal content	[58]
	SPF C57BL/6 mice ± probiotics **Dams** *n* = 10/group **Offspring** *n* = 8–10/group/time point (male and female)	*Akkermansia muciniphila* MucT (ATCC BAA-835) 1 × 10^8^–10^9^ CFU/day by gavage GD1–3 days before birth; PND3–PND21	Until PND42: No effect on body weight or intestinal tissue development	**Dams** No effect during pregnancy PND21: ↑ *A. muciniphila*, *Ruminococcus*_1, ↓ *Coriobacteriaceae* UCG-002, *Lachnospiraceae* UCG-001, *Ruminiclostridium* **Offspring** No effect on *A. muciniphila* abundance or ⍺-diversity PND42: Change in β-diversity, ↓ *Dubosiella*, *Lachnospiraceae*_UCG_001, *Parasutterella*, ↑ *Gordonibacter*, *Lachnospiraceae*_UCG_006, *Prevotellaceae*_UCG_001; ↑ the D-glutamine and D-glutamate microbial metabolism pathway	[98]
	Bama mini-sows ± probiotics or synbiotics **Dams** *n* = 16/group **Offspring** *n* = 8/group (male and female)	Mix: *Lactiplantibacillus plantarum* B90, *Saccharomyces cerevisiae* P11 Dose undisclosed, added in feed Synbiotic: probiotic mix + xylo-oligosaccharides (500 g/t feed) GD1–PND28	PND58 Probiotics: ↑ CAT, GPx and SOD activities in plasma, jejunum, and colon; ↓ malondialdehyde and H2O2 concentrations in plasma Synbiotics: ↑ CAT and SOD activities in plasma; ↑ glutathione, GPx activities and mRNA levels of antioxidant and mitochoindrial-related genes in jejunum; ↑ total antioxidant capacities in colon; ↓ colonic malondialdehyde	**Offspring** PND58 Probiotics: ↑ Bacteroidetes relative abundance in the jejunum and *Bifidobacterium* in the jejunum and colon. No change of total bacteria, *Bacteroides*, *Clostridium cluster* IV, *Lactobacillus* in jejunum or colon Synbiotics: ↑ Firmicutes, Bacteroidetes, *Bifidobacterium*, *Lactobacillus* (jejunum)	[69]
	Bama mini-sows ± probiotics or synbiotics **Dams** *n* = 16/group **Offspring** *n* = 8/group (male and female)	Mix: *Lactiplantibacillus plantarum* B90, *Saccharomyces cerevisiae* P11 Dose undisclosed, added in feed Synbiotic: probiotic mix + xylo-oligosaccharides (500 g/t feed) GD1–PND28	PND65 Probiotics: Improve immune response (Plasma: ↓ IL-2 and LPS concentrations, ↑ IgA; Jejunum: ↑ IL-10, interferon-α, sIgA; ileum: ↑ sIgA) ↑ jejunal villus height, villus height/crypt depth ratio Synbiotics: ↑ plasma IgA, ↑ jejunal IL-10, interferon-α, sIgA, ↑ ileal villus height	**Offspring** PND65: Effect on β-diversity, no effect on ⍺-diversity Probiotics: ↑ relative abundance of *Psychrobacter*, *Dialister*, *Oleomonas*, and *Facklamia* (ileum) Synbiotics: ↑ *Turicibacter*, SMB53, *Clostridium*, *Paracoccus*, *Thalassobius*, *Vibrio*, *Psychrobacter*, and *Blautia* (jejunum); ↑*Corynebacterium* and *Agrobacterium* (ileum)	[70]
	White Rex rabbit ± low /middle/high dose probiotics **Dams** *n* = 20/group **Offspring** *n* = 30/group (male and female)	*Clostridium butyricum* CCTCC AB: 2017089 1 × 10^3^ CFU/g (low dose), 1 × 10^4^ CFU/g (middle dose), 1 × 10^5^ CFU/g (high dose) in feed GD1–PND35, PND35–PND63 (offspring)	PND28: ↑ average daily weight gain PND63: ↑ α-amylase and chymotrypsin activity in SI; ↑ SI villi length and ↓ crypt depth; modulate SOD, GPx and CAT activity ↑ ZO-1, claudin, and occludin mRNA in SI High dose: ↓ IL-6, TNF-α and IFN-γ in ileum and colon; ↑ sIgA in duodenum. ↑ MyD88, TLR2, and TLR4 relative expression in SI	**Offspring:** PND63 Dose dependent effects High and middle dose: ↑ total bacteria abundance, ↑ Firmicutes; ↑ *Lactobacillus* & *Bifidobacterium*; ↑ *Clostridium cluster* IV, *Clostridium cluster* XIVa, and *Butyrivibrio fibrisolvens* in small intestine ↑ *Clostridium cluster* XIVa in colon Low dose: ↑ *Clostridium cluster* IV*, Clostridium cluster* XIVa*, Butyrivibrio fibrisolvens*, and *Lactobacillus* in duodenum	[99]
	C57BL/6 J mice ± probiotics **Dams** Undisclosed **Offspring** n = 9–10/group (male) n = 10–12/group (female)	*Lactococcus lactis* 5 × 10^5^ CFU/ml in drinking water GD10.5–PND1	PND1: ↑ the density of cortical neurons (Tbr1-expressing neurons, Satb2-expressing neurons in male), ↑ the density of blood vessels in the cortical plate Females: ↑ mitotic neural progenitor cell numbers 10 weeks: ↓ Anxiety-like behaviors Females: ↓ Freezing time in cue associated learning	Not assessed	[77]
	SPF Sprague Dawley rats ± probiotics **Dams** *n* = 4–5/group **Offspring** *n* = 12–14/group/time point (male) *n* = 11–14/group/time point (female)	*Lactobacillus helveticus* NS8 1 × 10^8^ CFU/ml in drinking water GD13–GD22	↓ body weight from PND51 until PND100-105 Antianxiety effect: PND35-40: Elevated plus maze: ↑ proportion of time in the open arms; PND35-40 & PND100-105: Open field test: ↑ proportion of the time/entries spent in the center and the distance travelled in the center, ↓ proportion of the latency to reach the center from the peripheral zone	Not assessed	[63]
	C57BL/6 mice + probiotic or fixed probiotic **Dams** *n* = 9/group **Offspring** *n* = 3–4/group/time point (male) *n* = 3–4/group/time point (female)	*Lacticaseibacillus rhamnosus* GG 1 × 10^8^ CFU/day (dams) by gavage, 1 × 10^7^ CFU/day (offspring) in feed GD18–birth, PND1–PND5 (offspring)	No impact on body weight PND21: ↑ crypt depth, villus height (jejunum, ileum, colon); 8 months: ↑ goblet cells, ZO-1, Claudin-1, and Occludin mRNA expression; ↑ ZO-1 and Claudin-3 protein expression ↑ SOD 1 & 2 mRNA expression, GPx expression	**Offspring** PND21: Strain detected in feces; no impact on ⍺-diversity, impact β-diversity; ↑ *Akkermansia muciniphila* and SCFA-producing bacteria (*Ruminococcus*, *Coprococcus*, *Odoribacter*, *Faecalibaculum*, and *Lachnospiraceae bacterium* A4) 8 months: Effect on β-diversity maintained ↑ *Lactobacillus, Parasutterella*, *Bifidobacterium*, *Akkermansia muciniphila*, ↓ *Oscillibacter*, *Escherichia*, *Ruminococcus*, *Helicobacter*, *Alistipes timonensis*	[59]
	C57BL/6 J mice + probiotic or fixed probiotic **Dams** Undisclosed **Offspring** *n* = 11–16/group (male) *n* = 10–13/group (female)	*Lacticaseibacillus rhamnosus* GG 1 × 10^8^ CFU/day (dams) by gavage, 1 × 10^7^ CFU/day (offspring) in feed GD18–birth; PND1–PND5 (offspring)	↑ BW at PND7, 14, 21, 28 PND21: ↑ Intestinal villus length, colonic crypt depth, cell proliferation and differentiation, tight junction proteins expression (ZO-1, claudin-3) 14 weeks (males): ↑ Fecal SCFA concentrations, ↓ anxiety-like behavior, ↑ colon epithelial growth factor receptor activation, serotonin transporter mRNA and protein levels; ↓ colon and serum 5-HT levels, ↑ brain-derived neurotrophic factor and ɣ-aminobutyric receptor	**Offspring** PND21: No effect on ⍺-diversity, impact β-diversity, ↑*Akkermansia*, ↓ *Helicobacter*, *Flavonifractor*, *Oscillibacter*, *Alistipes* ↓ bacterial pathways (motility proteins, flagellar assembly and porphyrin and chlorophyll metabolism) 14 weeks (male): No effect on ⍺-diversity, impact β-diversity, ↑ *Clostridium* XIVa, *Bifidobacterium* ↓ *Prevotella, Akkermansia*, *Bifidobacteria*	[62]
	Landrace × Yorkshire sows ± probiotics; offspring ± synbiotics (split-plot design) **Dams** 14–15/group **Offspring** *n* = 4–5/pen, 18–19 pens/group (male and female)	*Bacillus subtilis* C-3102 5 × 10^5^ (gestation), 1 × 10^6^ (lactation), 5 × 10^5^ (offspring) CFU/g of feed Prebiotics (MOS, offspring) GD30–PND19; PND19–PND42 (offspring)	No effect of nursery treatment on overall growth PND42: ↓ Weight in piglets born from probiotic supplemented sows	**Dams** GD30, GD113, PND18: ↑ Fecal *Bacillus subtilis* C-3102 & total Bacillus sp. **Offspring** PND18: ↑ Fecal *Bacillus subtilis* C-3102 & total *Bacillus* sp.	[111]
	Great Dane Bitches ± synbiotics (started at GD35 or GD56) **Dams** *n* = 5/group **Offspring** *n* = 30–32/group (male and female)	Mix: *Enterococcus faecium* DSM 10663, *Lactobacillus acidophilus* CECT 4529 3.36 × 10^8^ and 1.03 × 10^10^ CFU/10 kg of BW, respectively, in tablets Prebiotics (FOS: 480 mg/10 kg, MOS: 48.6 mg/10 kg of BW) GD35 or 56–birth	No effect on birth weight or litter parameters 9 weeks: ↓ Incidence of gastroenteritis 4-week supplementation had better results on first presentation of gastroenteritis	Not assessed	[112]
	Large White sows ± probiotics **Dams** *n* = 20/group **Offspring** *n* = undisclosed (male and female)	Mixes: *Bacillus subtilis* A + *Bacillus subtilis* B (BS-A + B); *Bacillus subtilis A* + *Bacillus licheniformis* (BS-A + BL); or *Bacillus subtilis B* + *Bacillus licheniformis* (BS-B + BL) Total of 4 × 10^9^ CFU/kg of feed GD85–PND21	PND21: ↑ Average weight and average daily gain at weaning BS-A + BL: ↑ number of weaned piglets	**Dams** GD100-112: No effect on *Bifidobacterium* spp., *Escherichia coli* or total bacteria BS-B + BL: GD112: ↑ *Clostridium cluster* IV PND7: ↓ Firmicutes	[60]
	Landrace x Yorkshire sows ± probiotics **Dams** *n* = 16/group **Offspring** *n* = 12/dams (male and female)	*Bacillus subtilis* PB6 4 × 10^8^ CFU/kg of feed GD90–PND21	↑ litter size, ↓ BW PND14: ↓ Serum cortisol concentration PND21: ↑ Litter weights and litter weight gains	**Dams** GD110: ↓ ⍺-diversity, ↑ *Gemmatimonadetes*, *Acidobacteria*, *Ruminococcaceae*_UCG-013 cc, ↓ Proteobacteria, Actinobacteria, *Streptococcus* relative abundance	[61]
	Pietrain sows ± probiotics **Dams** *n* = 10/group **Offspring** *n* = undisclosed (male and female, *n* = 5/group for hematological profile)	*Lactiplantibacillus plantarum* CAM6 1 × 10^10^ CFU/day, in fermented juice (pineapple, banana, and papaya peel) GD90–PND28	↓ Number of deaths before weaning ↑ body weight from PND7 until PND28 PND28: ↓ Diarrhea incidence, ↑ serum concentration of Na + , pCO2, and D-β-hydroxybutyrate, leukocytes, lymphocytes, and platelets in the piglets	Not assessed	[72]
	Large White × Landrace sows ± probiotics; offspring ± probiotics (split-plot design) **Dams** *n* = 12/group **Offspring** *n* = 10–6/group (male and female)	*Bacillus altitudinis* WIT588 4 × 10^9^ CFU/day (gestation), 1.2 × 10^10^ CFU/day (lactation), 1 × 10^9^ CFU/day (offspring) in feed GD100–PND26, PND26–PND54 (offspring)	PND21: No effect on body weight PND105, PND126: ↑ body weight PND34: Duodenum: ↑ villus length and area, crypt depth; Jejunum: ↑ crypt depth and area	**Dams** Strain detected in feces **Offspring** PND13: Strain detected in the feces of 12/20 piglets PND26: Strain detected in 16/20 piglets	[68]
	Landrace x Yorkshire sows ± low/high-dose probiotics **Dams** *n* = 5/group **Offspring** *n* = 55/group (male and female, n = 5/group for microbiota)	*Enterococcus faecium* DSM 7134 2.7 × 10^7^ CFU/kg (low-dose group), 5.4 × 10^8^ CFU/kg of feed (high-dose) GD101–PND21	↓ piglet pre-weaning mortality PND7: ↓ Diarrhea score PND21: ↑ Weaning weight, average daily gain and gain:feed ratio	**Offspring** PND21: ↑ Fecal *Lactobacillus* and *Enterococci*, ↓ *Escherichia coli* counts PND35: ↑ Fecal *Lactobacillus* and *Enterococci*	[71]
**Atopic diseases**					
	BALB/c mice exposed to ROFA or PBS on GD14, 16, 18 (air pollution exposure) Neonatal asthma model (ovalbumin sensitization) ± probiotics **Dams** Undisclosed **Offspring** *n* = 6–21/group (male and female)	*Bifidobacterium breve* M-16 1 × 10^9^ CFU/day in feed GD14–PND21	PND30: ↓ Eosinophils in the bronchoalveolar lavage fluid; ↓ allergic lung inflammation; ↓ mucus production; ↓ IL-5, IL-13, and Muc5ac gene expression	**Offspring** PND30: ↓ Firmicutes proportion ↑ *Gemella*, *Streptococcus*, *Dehalobacterium* & ↓ *Lactobacillus*	[81]
	BALB/c mice ± probiotics AD induced with MC903 or not (offspring, PND21) **Dams** *n* = 6/group **Offspring** *n* = 8/group (male and female)	*Limosilactobacillus reuteri* Fn041 or *Lacticaseibacillus rhamnosus* GG 1 × 10^9^ CFU/day by gavage GD14–PND21; PND21–PND28 (offspring)	PND28: Both strains: ↓ AD symptoms (skin swelling, mast cell, eosinophil infiltration), ↓ IL-4 and IL-12; *L. reuteri* Fn041: ↓ IgE, ↑ proportion CD4 + CD25 + Foxp3 + Tregs cells in mesenteric lymph nodes	**Offspring** PND28: *L. reuteri* Fn041: ↑ Actinobacteria, *Lactobacillus* and *Akkermansia*; LGG: ↓ ⍺-diversity; ↑ *Escherichia_Shigella*, *Enterococcus*; Both strains: ↓ *Alloprevotella*, *Klebsiella*, *Helicobacter*	[82]
**Inflammation**					
	C57BL/6 mice ± probiotics, DSS induced inflammation at PND70 **Dams** *n* = 2/group **Offspring** *n* = 4/group (male, microbiota) *n* = 15–16/group (female, weight loss–sample size microbiota undisclosed)	Mixes: *Limosilactobacillus reuteri* (strains 6798‐1, 6798‐jm, and 6798‐cm) or *Lactobacillus johnsonii* (strains 4901, 4903, 4931) 1 × 10^9^ CFU/ml by gavage GD12–birth; PND7–PND21 (every other day)	PND76: *L. reuteri*: ↓ post-DSS weight loss in females	**Offspring** Females: Both strains, PND21: Altered *Muribaculum* and *Ruminiclostridium* abundance PND30: Significant effect on β-diversity, altered *Lachnoclostridium*, *Muribaculum*, and *Tyzzerella* abundances *L. reuteri*, PND70: ↓ *Lachnoclostridium*, *Akkermansia*, *Parasutterella*, *Ruminiclostridium*, Bacteroides, ↑ *Lactobacillus*, *Muribaculum*, and *Bifidobacterium* Males: *L. reuteri*, PND70: ↓ *Alistipes* abundance, no effect on female altered genus	[73••]
	SPF C57BL/6 J mice ± probiotics, IL-1β induced postnatal systemic inflammation (PND14 and 28) **Dams** *n* = 5–6/group **Offspring** *n* = 3–9/group/time point (male and female)	Mix: *Lactobacillus acidophilus* ATCC 53544, *Bifidobacterium infantis* ATCC 15697 1 × 10^9^ CFU/day each GD16–PND21 Undisclosed	PND14: ↓ IL-1β-induced systemic levels of IL-6, KC, MCP-1, and IL-1β; normalize blood–brain-barrier permeability and occludin expression; regulate recruitment and extracellular matrix damage Promote neuronal development (↑ NeuN, NFL, and Syn1 expression) and oligodendrocyte progenitor cell development (↑ NG2 expression) PND28: No effect on IL-1β-induced ↑ of MCP-1 levels	Not assessed	[113]
**Obesity**					
	CD-1 IGS mice receiving a HFD or not started 6 weeks before mating until PND21, ± probiotics **Dams** *n* = 4/group **Offspring** *n* = 10–15/group/time point (male) *n* = 9–14/group/time point (female)	Mix: *Bacillus subtilis* PXN®21®, *Bifidobacterium bifidum* PXN® 23™, *Bifidobacterium breve* PXN® 25™, *Bifidobacterium infantis* PXN® 27™, *Bifidobacterium longum* PXN® 30™, *Lactobacillus acidophilus* PXN® 35™, *Lactobacillus delbruecki*i ssp. *bulgaricus* PXN® 39™, *Lactococcus casei* PXN® 37™, *Lactiplantibacillus plantarum* PXN® 47™, *Lacticaseibacillus rhamnosus* PXN® 54™, *Lactobacillus helveticus* PXN® 45™, *Ligilactobacillus salivarius* PXN® 57™, *Lactococcus lacti*s ssp. *lactis* PXN® 63™, *Streptococcus thermophilus* PXN® 66™ Total of 4 × 10^7^ CFU/ml in drinking water GD0.5–PND21	PND21: ↑ Female and male body weight; altered expression of gene involved in synaptic plasticity: restored HFD-induced ↑ of GLUN2C in female and modulate genes expression in control and HFD groups (GLUN2A, PFKB3, female GLUN2B, male SYP and CREB1) ↑ IL-6 prefrontal cortex expression in control and HFD, ↓ liver TLR4, and liver IL-1B in males PND21 & PND112: Prevent HFD-induced anxiety-like behavior ↑ gut propionate, butyrate, and brain lactate in control and HFD PND112: ↑ Brain-derived neurotrophic factor expression (BDNF, PFKB3, ΔFOSB and male cFOS and ZIF-268), ↑ female liver IL-6 in control and HFD	**Dams** PND21**:** ↑ Fecal *Lactobacillus* spp. in control and HFD, and ↑ *Bifidobacterium* spp. in control diet group	[79••]
	Sprague Dawley rats receiving HF diet or not from GD1 until PND21, ± probiotics **Dams** *n* = 3/group **Offspring** *n* = 7–8/group (male)	*Lacticaseibacillus casei* 2 × 10^8^ CFU/day by gavage GD1–PND21	12 weeks: No effect on body weight, Prevents HF-induced hypertension: ↓ systolic blood pressure from 6 to 12 weeks. ↓ acetate plasma levels; ↓ renal mRNA expression of Olfr78	**Offspring** 12 weeks Compared to control: ↑ *Parabacteroides* and *Akkermansia muciniphila*, ↓ Actinobacteria, *Bacteroides*, *Bacteroides*/*Prevotella*, Actinobacteria/Firmicutes ratio Compared to HF group: ↑ *Lactobacillus*, *Acholeplasma*, *Bacteroides acidifaciens*, ↓ *Alkaliphilus*, *Leptolyngbya*, *Prevotella albensis*, *Ruminococcus albus*	[95]
	Sprague Dawley rats receiving HFD or not from GD1 until PND21 ± probiotics **Dams** *n* = 3/group **Offspring** *n* = 8/group (male)	*Lacticaseibacillus casei* 2 × 10^8^ CFU/day by gavage GD1–PND21	16 weeks: Compared to HFD: No effect on BW, ↓ systolic blood pressure from 12 to 16 weeks, ↓ fecal propionate level, ↓ plasma TMAO levels and TMAO-to-TMA, ↑ DMA-to-TMAO conversion ratio, ↓ renal mRNA Ace expression	**Offspring** 3 weeks: Compared to HFD: No effect on ⍺- or β-diversity; Prevent the HF-diet induced ↓ *Alkaliphilus*, *Akkermansia* abundances and several *Lactobacillus* species 16 weeks: Prevents the ↓ *Lactobacillus*	[96]
	Wistar rats receiving HFHC diet or not from GD1 until PND21 ± probiotics **Dams** *n* = 5/group **Offspring** *n* = 5–10/group (male)	*Lactiplantibacillus plantarum* WJL 1 × 10^9^ CFU/day by gavage GD1–birth; PND2-21	↓ body weight and length from PND30 to PND90 ↓ blood pressure and recovered vascular function in later life	**Dams** Restore ⍺-diversity but not β-diversity No effect on dyslipidemia induced dysbiosis ↑ *Lachnospiraceae* (*Ruminococcus*, *Blautia*, *Dorea*, and *Coprococcus*)	[94]
**Neurodegenerative disease**					
	Wistar Albino rats receiving intraperitoneal injection of LPS (100 μg/kg) on GD17 (neuroinflammation) ± probiotics **Dams** *n* = 5/group **Offspring** *n* = 18/group (male and female)	*Ligilactobacillus salivarius* ATCC 11741, or *B. bifidum* ATCC 29521, or both Total of 4 × 10^9^ CFU/ml by gavage GD1–birth	PND1: Improved brain APP levels and mRNA, gamma-secretase and beta-secretase levels, improved brain BDNF mRNA expression ↓ brain damage in the cortical area	Not assessed	[114]
	Sprague Dawley rats exposed to lead one week after mating until offspring sacrifice (memory dysfunction model) ± probiotics **Dams** *n* = 3/group **Offspring** *n* = 6–10/group (female)	Mix: *Bifidobacterium longum* BL986, *Lactobacillus acidophilus* LA1063, *Limosilactobacillus fermentum* LF26, *Lactobacillus helveticus* LH43, *Lacticaseibacillus paracasei* LPC12, *Lacticaseibacillus rhamnosus* LRH10, *Streptococcus thermophilus* ST30 Total of 1 × 10^10^ CFU/day in drinking water GD7–PND21, PDN21–PND68 (offspring)	PND22 & PND68: Reversed lead-led memory impairment and loss of hippocampal spines PND68: Restore IL-6 levels and the epigenetic event H3K27me3 level in the hippocampus	**Offspring** PND68**:** Restore ⍺-diversity, Firmicutes/Bacteroidetes ratio, Proteobacteria, Actinobacteria. ↑ *Helicobacter*, *Bifidobacterium*, *Bacteroides*, ↓ *Anaerovibrio*, *Ruminococcaceae*_UCG-008, *Lactobacillus*	[100]

*AD* atopic dermatitis, *BMI* body mass index, *BW* birth weight, *CAT* catalase, *CFU* colony forming units, *FOS* fructo-oligosaccharides, *GD* gestational day, *GPx* glutathione peroxidase, *HDL* high-density lipoprotein, *HF* high-fructose, *HFD* high-fat diet, *HFHC* high-fat and high-cholesterol, *Ig* immunoglobulin, *IL* interleukin, *LDL* low-density lipoprotein, *LPS* lipopolysaccharides, *MOS* mannan-oligosaccharides, *MUC2* mucin 2, *PND* postnatal day, *ROFA* residual oil fly ash, *SCFA* short-chain fatty acids, *SI* small intestine, *SOD* superoxide dismutase, *SPF* specific pathogen-free, *ZO-1* zonula occludens 1

**Table 2:** Clinical studies evaluating probiotic administration starting before and during gestation on offspring outcomes

Time of administration	Population characteristics	Probiotic, dose, vehicle	Infant outcomes	Effect on maternal and infant gut microbiota	Ref
*Before pregnancy*					
**Healthy population**					
	30.53 ± 3.40 years old women, Singapore, New Zealand, UK, *n* = 585	Mix: *Lacticaseibacillus rhamnosus* NCC 4007, *Bifidobacterium animalis* NCC 2812 1 × 10^10^ CFU/day each, in powder Myo-inositol (4 g/day), Vitamin D (10 ug/day), Riboflavin (1.8 mg/day), Vitamin B6 (2.6 mg/day), Zinc (10 mg/day) From preconception–birth	No effect on BW ↓ number of preterm births	Not assessed	[115]
*During pregnancy*					
**Healthy population**					
	24.8 ± 5.3 years old women, Philippines, *n* = 208	Mix: *Bifidobacterium lactis* CNCC I-3446, *Lactobacillus rhamnosus* CGMCC 1.3724 1.4 × 10^9^ CFU/day each, in powder Nutritional supplement (proteins, carbohydrates, fats, vitamins and minerals) 24–28 weeks of gestation–2 months after birth	No effect on diarrhea incidence 12 months: ↑ weight, height, and weight-for-age z-score	Not assessed	[116]
	29.97 ± 3.84 years old women, Norway, *n* = 278	Mix: *Lacticaseibacillus rhamnosus* GG, *Bifidobacterium animalis* subsp*. lactis* Bb-12, *Lactobacillus acidophilus* La-5 5 × 10^10^, 5 × 10^10^, 5 × 10^9^ CFU/day, respectively, in fermented milk 36 weeks of gestation–3 months after birth	40% reduction in AD among 2-year-old children (ProPACT) Association of probiotic effect on AD with intrinsic infant gut microbiota	**Infants** No effect on ⍺-diversity. Effect on β-diversity at 10 days between probiotic-treated infant developing AD or not. ↑ *Bifidobacterium dentium* in children developing AD in the probiotic group	[83]
	31 ± 3 years old women, Norway, *n* = 140	Mix: *Lacticaseibacillus rhamnosus* GG, *Bifidobacterium animalis* subsp. *lactis* Bb-12, *Lactobacillus acidophilus* La-5 5 × 10^10^, 5 × 10^10^, 5 × 10^9^ CFU/day, respectively, in fermented milk 36 weeks of gestation–3 months after birth	↓ risk of AD following probiotic supplementation (ProPACT) Peripheral blood regulatory T cells at 3 months: ↓ proportion of Th22 cells; No effect on Th1, Th2, Th9, Th17 Probiotic effect partially mediated through the reduction in Th22 cells	Not assessed	[84]
	29.6 ± 3.9 years old women, Norway, *n* = 298	Mix: *Lacticaseibacillus rhamnosus* GG, *Bifidobacterium animalis* subsp*. lactis* Bb-12, *Lactobacillus acidophilus L*a-5 5 × 10^10^, 5 × 10^10^, 5 × 10^9^ CFU/day, respectively, in fermented milk 36 weeks of gestation–3 months after birth	Prevention of allergy (ProPACT)–early life gut mycobiota and maternal-offspring transfer No effect on offspring gut mycobiota	Not assessed	[117]
	27.2 ± 3.2 years old women, China, *n* = 30	Mix: *Bifidobacterium longum*, *Lactobacillus delbrueckii bulgaricus*, *Streptococcus thermophilus* 2 × 10^7^, 2 × 10^6^, 2 × 10^6^ CFU/day, respectively, in tablets 32 weeks of gestation–birth	No effect on birth anthropometrics	**Mothers** No effect on ⍺- or β-diversity; 48 altered OTUs compared to control; ↓ Clostridiales, Clostridium_sensu_stricto, and *Holdemanella*; ↑ *Porphyromonadaceae*, *Holdemanella*, and *Lachnospiraceae*	[52]
	27.2 ± 1.16 years old women, China, *n* = 25	Mix: *Bifidobacterium*, *Lactobacillus* and *Streptococcus* (species undisclosed) 1 × 10^7^, 1 × 10^6^, 1 × 10^6^ CFU/day, respectively, in tablets 32 weeks of gestation–birth	No effect on birth anthropometrics	Not assessed	[53]
	20–29 years old women, Indonesia, *n* = 70	*Bifidobacterium animalis* subsp. *lactis* HNO 19 1 × 10^9^ CFU/day in capsules 36 weeks of gestation–3 months after birth	No effect on infant gut mucosal integrity at birth and 3 months (urine IFABP, fecal ⍺-1-antitrypsin and calprotectin levels)	Not assessed	[118]
	Women aged 16 years old or older (29.0 ± 5.6), United Kingdom, n = 422	Mix: *Lactobacillus salivarius* CUL61, *Lactobacillus paracasei* CUL08, *Bifidobacterium animalis subsp. lactis* CUL34, *Bifidobacterium bifidum* CUL20 1 × 10^10^ CFU/day in capsules 36 weeks of gestation–birth; birth–6 months (infants)	5 years: No protection against asthma outcomes or eczema	Not assessed	[86]
	18–49 (28.59 ± 5.3) years old women, Iran, *n* = 175	*Limosilactobacillus reuteri* LR92 DSM 26866 1 × 10^8^ CFU/day in oil droplets 36 weeks of gestation–birth	↓ colic frequency and severity until 5 months No impact on BMI; No effect on feeding pattern	Not assessed	[54•]
**At risk for allergic diseases**					
	29.5 (26.2–32.7) years old women, with allergy in the family; Finland, *n* = 15	Mix: *Lacticaseibacillus rhamnosus* GG, *Bifidobacterium animalis* subsp*. lactis* Bb12 1 × 10^9^ CFU/day in capsules 4 weeks of gestation–Birth	No effect on birth weight and height, and weight and height at 1 and 6 months	Not assessed	[56•]
	34 (30–36) years old women, history of treated asthma, eczema, or hay fever; New-Zealand, *n* = 423	*Lacticaseibacillus rhamnosus* HN001 6 × 10^9^ CFU/day in capsules 14–16 weeks of gestation–6 months after birth	No effect on eczema, wheeze, or atopic sensitization at 12 months	Not assessed	[90•]
	34 (30–36) years old women, history of treated asthma, eczema, or hay fever; New-Zealand, *n* = 373	*Lacticaseibacillus rhamnosus* HN001 6 × 10^9^ CFU/day in capsules 14–16 weeks of gestation–6 months after birth	No effect on birth anthropometrics (BW, BL, BMI, head circumference)	Not assessed	[55]
	18–45 (29) years old women, family history of allergic diseases, Sweden, *n* = 88	*Limosilactobacillus reuteri* DSM 17938 2 × 10^9^ CFU per day, in oil droplets ω-3 PUFA (3.84 g/day) 20 weeks–birth	No effect on BW	Not assessed	[119]
	32.0 (29.0–33.0) years old women, family history of allergic diseases, Sweden, *n* = 63	*Limosilactobacillus reuteri* DSM 17938 2 × 10^9^ (mothers), 1 × 10^8^ CFU/day (infants), in oil droplets ω-3 PUFA (3.84 g/day) 20 weeks–birth; probiotics: birth–12 months (infants); ω-3: birth–6 months (mothers)	All treatments: ↑ hypermethylation of cord blood CD4 + T cells Probiotics + ω-3: ↑ number of differentially methylated CpG sites (immune-related pathways)	Not assessed	[109•]
	Women of undisclosed age; mother or father diagnosed with allergy disease; Finland, *n* = 422	Mix: *Bifidobacterium breve* Bb99, *Propionibacterium freundenreichii subsp. shermanii* JS, *Lactobacillus rhamnosus* Lc705, *Lactobacillus rhamnosus* GG 2 × 10^8^, 2 × 10^9^, 5 × 10^9^, 5 × 10^9^ CFU/day, respectively, in capsules GOS (infant, 0.8 g/day) 36 weeks–birth; birth–6 months (infants)	Not assessed	**Infants**: 3 months: ↑ *Bifidobacterium breve*, *Lacticaseibacillus rhamnosus* ↓ antibiotic & caesarian-associated changes Probiotic effect dependent on infant diet: Breastfed: ↑ *Bifidobacteria*, ↓ Proteobacteria and Clostridia	[101]
	Women of undisclosed age; mother or father diagnosed with allergy disease; Finland, *n* = 807	Mix: *Lacticaseibacillus rhamnosus* GG, *Lacticaseibacillus rhamnosus* LC705, *Bifidobacterium breve* Bb99, *Propionibacterium freudenreichii* ssp. *shermanii* JS 1 × 10^10^, 1 × 10^10^, 4 × 10^8^, 4 × 10^9^ CFU/day, respectively, in capsules GOS (infant, 0.8 g/day) 36 weeks of gestation–birth; birth–6 months (infant)	10 years: No effect on lifetime prevalence of any allergic disease. ↓ Lifetime prevalence of eczema and food allergy Between 5 and 10 years: ↑ allergic rhino-conjunctivitis in vaginally delivered children; ↓ upper respiratory tract infections in caesarean-delivered children	Not assessed	[88]
	Women of undisclosed age, mother or father diagnosed with allergy disease; Finland, *n* = 642	Mix: *Lacticaseibacillus rhamnosus* GG, *Lacticaseibacillus rhamnosus* LC705, *Bifidobacterium breve* Bb99, *Propionibacterium freudenreichii* ssp. *shermanii* JS 1 × 10^10^, 1 × 10^10^, 4 × 10^8^, 4 × 10^9^ CFU/day, respectively, in capsules GOS (infant, 0.8 g/day) 36 weeks of gestation–birth; birth–6 months (infant)	13 years: no differences in the prevalence rate of doctor-diagnosed allergic disease or allergic disease with IgE sensitization; ↑ inhalant-specific IgE sensitization Caesarean-delivered subgroup: ↓ incidence of allergy and eczema	Not assessed	[89]
	Women with family history of eczema, asthma, gastrointestinal allergy, allergic urticaria or allergic rhino conjunctivitis; Sweden, *n* = 188	*Limosilactobacillus reuteri* ATCC 55730 1 × 10^8^ CFU/day in oil droplets 36 weeks of gestation–birth (mother) & 1–3 days–12 months (infants)	↓ DNA methylation of CD4 + T cells genes related to immune maturation and allergy development at birth	Not assessed	[85]
	Women of undisclosed age; mother or father diagnosed with allergy disease; New-Zealand, *n* = 342	Either *Lacticaseibacillus rhamnosus* HN001 or *Bifidobacterium animalis subsp. lactis* HN019 6 × 10^9^ and 9 × 10^9^ CFU/day, respectively, in capsules 35 weeks of gestation–birth; birth–2 years (infant)	11 years HN001: ↓ Lifetime prevalence of eczema, atopic sensitization, wheeze; ↓ in 12-month prevalence of eczema HN019: no effect	Not assessed	[87]
	Women of undisclosed age; mother or father diagnosed with allergy disease; New-Zealand, *n* = 342	Either *Lacticaseibacillus rhamnosus* HN001 or *Bifidobacterium animalis* subsp. *lactis* HN019 6 × 10^9^ and 9 × 10^9^ CFU/day, respectively, in capsules 35 weeks of gestation–birth; birth–2 years (infant)	11 years Both strains: No effect on cognitive, behavioral and mood outcomes	Not assessed	[78]
**Obesity**					
	28.8 ± 5.7 years old women, BMI ≥ 30, New Zealand, *n* = 230	Mix: *Lacticaseibacillus rhamnosus* GG, *Bifidobacterium subsp. lactis* BB12 6.5 × 10^9^ CFU/day in capsules 12 weeks–17 weeks of gestation	No effect on BW No effect on infant anthropometrics, admission to NICU, and composite neonatal morbidity	Not assessed	[47]
	30.8 ± 4.8 years old women, BMI ≥ 25, Finland, *n* = 439	Mix: *Lacticaseibacillus rhamnosus* HN001, *Bifidobacterium animalis* ssp. *lactis* 420 1 × 10^10^ CFU/day each in capsules Fish oil capsules (2.4 g of n-3 fatty acids, with 1.9 g docosahexaenoic acid, 0.22 g eicosapentaenoic acid) 13 weeks of gestation–6 months	No effects on birth anthropometrics	Not assessed	[120]
	> 18 years old women (30.7 ± 4.5), BMI ≥ 30 and < 35, Denmark, *n* = 49	Mix: *Streptococcus thermophilus* DSM 24731, *Bifidobacterium breve* DSM 24732, *Bifidobacterium longum* DSM 24736, *Bifidobacterium infantis* DSM 24737, *Lactobacillus acidophilus* DSM 24735, *Lactiplantibacillus plantarum* DSM 24730*, Lacticaseibacillus paracasei* DSM 24733, *Lactobacillus delbrueckii* subsp*. bulgaricus* DSM 24734 Total of 4.5 × 10^10^ CFU/day in capsules 14–20 weeks of gestation–birth	No effect on BW and BL, LGA or SGA	**Mothers** ↑ in ⍺-diversity and β-diversity from baseline to birth; ↑ abundance of *Bifidobacterium*, *Lactobacillus*, *Streptococcus salivarius* with time	[48]
	> 18 years old women (31.3 ± 4.7), BMI ≥ 25, Australia, *n* = 411	Mix: *Lacticaseibacillus rhamnosus* GG, *Bifidobacterium animalis* spp. *lactis* BB-12 1 × 10^9^ CFU/day in capsules 20 weeks of gestation–birth	↓ SGA incidence, no effect on other birth outcomes (preterm, hypoglycemia, macrosomia, LGA, BW, % fat)	Not assessed	[92]
	> 18 years (29.5 ± 6.2) years old women, BMI ≥ 25, Iran, *n* = 126	Mix: *Lactobacillus acidophilus* La5, *Bifidobacterium animalis* spp. *lactis lactis* Bb12 1 × 10^10^ and 1 × 10^9^ CFU/day, respectively, in yoghurt 24 weeks of gestation–birth	↓ bilirubin levels at 3–5 days, ↓ treatments rate No effect on infant anthropometrics	Not assessed	[49]
**GDM**					
	18–40 years old (32.03 ± 5.53) women, at high-risk of GDM, Iran, *n* = 507	Mix: *Lactobacillus acidophilus* LA1, *Bifidobacterium longum* sp54, *Bifidobacterium bifidum* sp9 7.5 × 10^9^, 1.5 × 10^9^, 6 × 10^9^ CFU/day, respectively, in capsules 14 weeks of gestation–24 weeks	No effect on BW and macrosomia	Not assessed	[50]
	18–45 years old (32.50–5.02) women with the diagnosis of GDM, Thailand, *n* = 57	Mix: *Lactobacillus acidophilus, Bifidobacterium bifidum* (strain undisclosed) 1 × 10^9^ CFU/day, respectively, in capsules 24–27 weeks to 28–31 weeks of gestation	No effect on BW or neonatal hypoglycemia	Not assessed	[51]
	31.64 ± 5.97 years old women with the diagnosis of GDM, Iran, *n* = 84	Mix: *Lactobacillus acidophilus, Bifidobacterium lactis* (strain undisclosed) Total of 1 × 10^6^ CFU/day in yoghurt For 8 weeks during the last trimester	↓ BW; ↓ macrosome neonates; No change of BL, head circumference or GA	Not assessed	[121]

*AD* atopic dermatitis, *BL* birth length, *BMI* body mass index, *BW* birth weight, *CFU* colony forming units, *GA* gestational age, *GDM* gestational diabetes mellitus, *GOS* galacto-oligosaccharides, *IFABP* intestinal fatty-acid binding protein, *LGA* large for gestational age, *OTU* operational taxonomic units, *PUFA* polyunsaturated fatty acids, *SGA* small for gestational age

## Probiotics in the Context of the Developmental Origins of Health and Disease

The Developmental Origins of Health and Disease (DOHaD) paradigm is based on the principle of developmental plasticity, which refers to the phenomenon by which “a given genotype can give rise to a range of different physiological or morphological states in response to various environmental exposures throughout development.” The term programming refers to a stimulus introduced at a “critical” or “sensitive” period, which causes long-term consequences for an organism [25]. A key principle of DOHaD is the existence of “windows of opportunity”, i.e., the prenatal stages of life (from pre-conception to embryonic and fetal stages and birth), infancy, and adolescence [25]. These time-sensitive life stages of exposure lead to tissue-specific effects thought to optimize or alter one’s biological potentials, and promote long-term health or a disease state [26, 27]. For example, undernutrition during pregnancy induces structure and functional remodeling in the fetus preserving brain development and prioritizing survival, negatively impacting development of other functions such as glucose metabolism and insulin sensitivity [26]. With time, as the evolutionary advantage of developmental plasticity begins to decrease, an individual’s ability to adapt to positive or negative environmental challenges becomes more limited [26]. Historically, the area of DOHaD research has primarily focused on overnutrition or undernutrition, linked with the manifestation of non-communicable diseases in later life, such as obesity or diabetes mellitus. Prenatal and early infancy exposures have been specifically studied at the macro- and micro-nutrient levels, with offspring outcomes ranging from glucose homeostasis to blood pressure [28]. In utero epigenetic modifications may be underlying mechanisms [29]. The gut microbiota has been proposed to regulate host gene expression epigenetically [30, 31], for example, via DNA methylation [32] with implications for offspring disease susceptibility [33]. The intestinal microbiota is a dynamic and interactive across-kingdom ecosystem composed of characteristic microbial communities co-evolving with their host [34]. While what constitutes a health-compatible microbiota remains elusive [35], many taxa have been identified whose altered representation is associated with diseases. Specifically, in infancy, microbiota variation is a predictor of overweight [5], asthma, and allergy [3]. The microbiota is seeded by the maternal microbiota [36] and continues to develop during infancy [37] thus going through sequential stages of plasticity that encompass pregnancy and are susceptible to programming, while being a determinant of life-long health [38]. In this context, maternally administered probiotics can be used as a dietary intervention that targets the offspring intestinal ecosystem. In utero and early life exposure to probiotics may affect growth and gut health [39–41], suggesting that specific windows of opportunities may exist for probiotic administration.

### Growth and Anthropometric Indices at Birth

Probiotics have a long history of use to support growth of farm animals [42, 43]. Investigation into the underlying mechanisms suggests that they play a role in hormone metabolism. For example, certain *Lactiplantibacillus plantarum* strains support *Drosophila melanogaster* larval growth via target of rapamycin (TOR)-dependent mechanisms and hormonal growth signaling [44]. *Lactiplantibacillus plantarum* strains also support systemic growth in undernourished mice via growth hormone sensitivity enhancement and increased tissue insulin-like growth factor 1 activity [45]. Previous studies investigating the impact of maternal probiotics intake on offspring growth have largely focused on metabolic or allergic diseases as main outcomes [39, 46]. In recent studies in overweight women and women diagnosed with GDM, mid- or late-pregnancy supplementation with several mixtures of *Lactobacillus*, *Bifidobacterium*, and *Streptococcus* species had no effect on infant anthropometrics at birth [47–51]. Recently, several studies were also completed in healthy participants. As shown in Table 2, supplementation with various probiotic mixes starting at the beginning of pregnancy, at the second trimester, or at the end of pregnancy had no impact on neonatal birth weight, body mass index, birth length, femur length, or head circumference [52, 53, 54•, 55, 56•]. These findings align with a recent meta-analysis investigating infant birth weight following exposure to *Lacticaseibacillus rhamnosus* strains alone or in mixtures with *Streptococcus* or *Bifidobacterium* strains started during mid- or late-pregnancy [57]. Sex-specific effects have not been comprehensively studied.

Birth anthropometrics are important prognostic markers of healthy growth. Long-term effects of maternal probiotics on offspring growth during infancy and until adulthood have not been investigated in clinical studies; however, some studies have been conducted in animals. In healthy rodent models, the probiotic strains *Limosilactobacillus fermentum* CECT5716 and *Lacticaseibacillus rhamnosus* GG supplemented during pregnancy and lactation did not impact offspring body weight at weaning [58, 59]. Though supplementation with *Bacillus subtilis* PB6 or A and B from late pregnancy until weaning led to increased body weight in healthy piglet models (male and female combined) [60, 61]. Similarly, maternal intake of *Lacticaseibacillus rhamnosus* GG from late pregnancy until birth, combined with neonatal administration during the first five days of life, increased offspring weight during the four weeks after birth compared to the control mice that received the inactivated strain [62]. Findings were combined from male and female piglets. Interestingly, supplementation with *Lactobacillus helveticus* NS8 at the end of gestation in a healthy rat model led to a decreased body weight from late adolescence (51 days of life) until adulthood (76 and 86 days of life), without influencing the weight difference between males and females [63]. These findings suggest that the duration of the follow-up measurements is an important consideration to fully determine the effect of maternal probiotics exposure on offspring growth.

### Intestinal Barrier and Gut Health

The intestinal barrier is critical to host health and a recognized target for preventative and therapeutic strategies [64]. The gut microbiota contributes to the protection of the epithelium from luminal pathogens and antigens, both physically and chemically [65], and supports the development of the infant immune system [3]. The intestinal barrier and the microbiota co-evolve in early life and reciprocally influence each other, resulting in the establishment of a mature intestinal ecosystem [66]. Probiotics have long been recognized to sustain the intestinal barrier, including in early life [40, 41]. Additionally, studies have proposed that the intestinal barrier, including the tight junction and the toll-like receptor associated pathways, is under epigenetic regulation [33]. Interestingly, in piglets, a recognized relevant model for pediatric nutritional studies assessing intestinal outcomes [67], maternal probiotics supplementation with *Bacillus altitudinis* WIT588 starting during late pregnancy increases intestinal crypt depth and villus length at postnatal day 34 [68]. In mice, supplementation with *Lacticaseibacillus rhamnosus* GG increases cell proliferation and differentiation and tight junction protein expression at postnatal day 21 [59, 62]. These findings suggest that maternal probiotic supplementation supports offspring intestinal digestive and absorptive functions in infancy [69]. Sex effects were not examined in these studies.

The long-term impact of this *Lacticaseibacillus rhamnosus* GG supplementation was investigated at 8 months of age when mice displayed increased goblet cell numbers and tight junction gene expression [59]. This was accompanied by increased antioxidant enzyme activities [59], and this increase was also demonstrated in the adult progeny of mini-sows that had received a *Lactiplantibacillus plantarum* B90 and *Saccharomyces cerevisiae* P11 mix, alone or with xylooligosaccharides, since the beginning of gestation [69]. This enhanced antioxidant capacity may help reduce ageing-associated oxidative stress and was associated with an increased jejunal villus height at 65 days [70]. Overall, findings from these pre-clinical studies suggest that maternal probiotic supplementation may help maintain gut barrier integrity in early life and adulthood and may improve antioxidant status. Indeed, recent studies support preventative effects of maternally administered probiotics in offspring with impaired intestinal barrier. In sows, maternal intake of *Enterococcus faecium* DSM7134 and *Lactiplantibacillus plantarum* CAM6 was shown to reduce diarrhea incidence in offspring [71, 72]. In a clinical trial, *Limosilactobacillus reuteri* LR92 supplementation to healthy mothers starting at late pregnancy and until birth led to a decreased colic frequency and severity in infants until 5 months of age, although the influence of sex on this effect was not reported [54•]. Interestingly, a recent study in mice showed that maternal administration of a mix of three *Limosilactobacillus reuteri* strains protected female offspring from dextran sodium sulfate-induced colitis [73••], although no protective effects were seen in male offspring. Taken together, these findings suggest that administration of selected probiotics during pregnancy may beneficially prevent intestinal inflammation in the offspring. More studies are required to understand sex-differences in responses and underlying mechanisms.

### Neurodevelopment and Anxiety-Like Behavior

The gut ecosystem, including the microbiota, and the peripheral and central nervous system entertain a continuous bi-directional communication that is typically referred to as the gut-brain axis. Underlying mechanisms include endocrine pathways through cortisol and the hypothalamic–pituitary–adrenal axis, immunomodulation through cytokines, and interaction with the vagus nerve and the enteric nervous system [74]. The effects of probiotics on the gut-brain axis have been recently reviewed; Mörkl et al. demonstrated that probiotics may be therapeutically beneficial in the context of depression, but not schizophrenia, while data for anxiety are lacking [74]. Interestingly, these conditions may be rooted into neurodevelopment in early life and environmental exposures during stages of plasticity [75]. Moreover, the gut microbiota has been shown to foster fetal thalamocortical axonogenesis [76]. Thus, probiotic exposure during early stages of life, including in utero, may have preventative potential. In fact, one study in mice found that maternal supplementation of *Lactococcus lactis* (strain not disclosed) from 10.5 days of gestation increased blood vessel numbers and size in the cortical plate and cortical neurons density in offspring of both sexes at postnatal day 1, while the numbers of mitotic neural progenitor cells were increased in females only [77]. Interestingly, effects were investigated in the context of anxiety, which is known to appear early among psychiatric disorders [75]. At 10 weeks of age, females but not males from the above study had a higher activity level in bright zones and reduced fearful behavior [77]. Similarly, adolescent offspring, especially females, of rats that received *Lactobacillus helveticus* NS8 in late pregnancy spent more time in open spaces compared to controls [63]. No effects on cognition and behavior were seen in 11 years old children born at risk for allergic disease and exposed to *Lacticaseibacillus rhamnosus* HN001 or *Bifidobacterium animalis* subsp. *lactis* HN019 since late gestation and until 2 years of age [78]. Interestingly, these benefits of probiotics are also seen in the context of obesity. Administration of a multi-strain probiotic to mouse dams since conception prevented obesity-induced anxiety-like behavior in offspring at weaning and at adulthood in both sexes [79••]. Thus, findings from these recent pre-clinical studies suggest that maternal probiotic intake may reduce anxiety-like behavior in the next generation, potentially through modulation of neurodevelopment processes in utero and early life. Moreover, these studies suggest that effects may be sex-specific. Since psychiatric disorders manifest differently between sexes and are more prevalent and damaging in females compared to males [80], more studies should be conducted to understand if maternally administered probiotics hold potential for sex-targeted clinical applications.

### Allergic Diseases

The effect of probiotic supplementation during prenatal and early life on prevention of allergic diseases has been extensively studied, leading to the release of recommendations for populations at risk [7]. These guidelines are the same for both sexes, and previous studies largely focused on *Lactobacillus* species, alone or in combination with *Bifidobacterium*. Recent literature has continued investigating the mechanisms behind probiotic preventative effects. Studies in mice show that probiotics, typically administered starting at mid gestation, act through immune-mediated mechanisms (details in Table 1), in both models of asthma [81] and atopic dermatitis [82], and these effects may be strain-specific [82].

Interestingly, studies in healthy women receiving probiotics from 36 weeks of gestation to 3 months of lactation showed a reduction in infant atopic dermatitis incidence, which was associated with decreased proportion of Th22 but independent from Th1/Th2 balance [83, 84]. A clinical study of women with a family history of treated allergic diseases receiving *Limosilactobacillus reuteri* ATCC 55730 during late pregnancy reported a modulation in DNA methylation of CD4 + T cells genes related to immune maturation and allergy development in infants at birth, suggesting that epigenetic modifications may mediate the preventative effects of probiotics in this context [85]. Further studies are needed to decipher the exact mechanisms. Long-term effects were also investigated in clinical studies. While combined maternal and infant supplementation with a probiotic mix (*Lactobacillus salivarius* CUL61, *Lactobacillus paracasei* CUL08, *Bifidobacterium animalis* subsp. *lactis* CUL34, *Bifidobacterium bifidum* CUL20) had no effects on asthma or eczema prevalence in 5-year-old children [86], the administration of another probiotic mix (*Lacticaseibacillus rhamnosus* GG and LC705, *Bifidobacterium breve* Bb99, *Propionibacterium freudenreichii* ssp. *shermanii* JS) or of *Lacticaseibacillus rhamnosus* HN001 to the mother and infant decreased the lifetime prevalence of eczema and atopic sensitization [87, 88] and food allergy [88] in 10- and 11-year-old children, respectively. Probiotic preventive effects on upper respiratory tract infections [88] and eczema [89] were also reported in populations subgroups of caesarean-delivered infants, after 10 and 13 years, respectively. Of importance, *Lacticaseibacillus rhamnosus* HN001 was found not to have the same effects on infant eczema, wheeze, and atopic sensitization when given to the mother alone without infant supplementation [87, 90•], thus calling for more studies investigating the timing of probiotic administration.

### Metabolic Disorders

Maternal nutritional status is a determinant of offspring metabolic health and both pre-clinical and clinical studies demonstrate that various components of the metabolic syndrome are susceptible to fetal programming [91]. The study of maternal probiotics administration and effects on offspring metabolic health is still in its infancy, with most human studies focused solely on maternal outcomes [47–49, 92]. In pre-clinical studies, offspring have been evaluated at weaning and adulthood, with hypertension and risk for cardiovascular disease being the most studied conditions [93–96]. Interestingly, the only pre-clinical study that we found administering probiotic since pre-conception identified sex- and time-specific (weaning versus young adults) benefits in offspring and stronger evidence for reduced cardiovascular dysfunction in female offspring [93]. Most other pre-clinical studies have been conducted in males only (Table 1).

A study in normal-weight women supplemented with *Lacticaseibacillus rhamnosus* GG and *Bifidobacterium lactis* Bb-12 from the 4th week of pregnancy until birth showed a reduction in DNA methylation of obesity and weight gain-related genes in offspring, suggesting again that epigenetics might be a mechanism underlying probiotics programming effects [56•]. As pre-clinical studies revealed that significant weight differences are observable at weaning and adulthood, although males and females were not studied separately, it would be interesting to assess whether maternal probiotics effects on offspring growth are also observable during infancy and puberty, and in a sex-specific manner.

### Intestinal Microbiota

Modulation of the microbiota is not a prerequisite for probiotics effects. In addition, resilience of an established gut microbiota makes it difficult to modify using exogenous microorganisms, though microbiota susceptibility is likely higher during early stages of plasticity. A recent meta-analysis found that maternal exposures including body mass index, drug-induced alteration of the microbiota, and probiotics administration influence the offspring intestinal microbiota [97]. In animal models, maternal probiotic supplementation had different effects on the offspring gut microbiota composition and structure, depending on the species administered and the time of administration [58, 59, 62, 69–71, 73••, 81, 82, 93, 95, 96, 98–100]. Different effects were also observed in various regions of the intestinal tract [69]. Interestingly, co-administration of probiotic *Lactiplantibacillus plantarum* B90 and *Saccharomyces cerevisiae* P11 with prebiotic xylooligosaccharides modified the probiotic effect on microbiota outcomes of piglets [69]. Probiotics with and without prebiotic increased Bacteroidetes and *Bifidobacterium* jejunal relative abundance, while only co-administration also increased Firmicutes and *Lactobacillus* [69]. Most human studies of maternally administered probiotics did not assess infant microbiota. For those that did, maternal probiotic intake was found to transiently increase the relative abundance of administered probiotic species or strain in the offspring [83, 101], regardless of the participants being healthy or at risk for allergic diseases (Table 2). Pre-clinical studies are also the only studies assessing microbial outcomes long-term, including effects on microbial diversity and/or composition at puberty in healthy mice [98] and mice born to dams exposed to an obesogenic diet [93], as well as at young adulthood [93, 95, 96]. Interestingly, some of these studies investigated microbiota outcomes in offspring of both sexes. This is relevant because there is evidence for sex-specific microbiota taxa as early as 2 weeks after birth [102], which likely become more pronounced during puberty [103]. Some of the probiotics studied showed sex-dependent effects on microbiota structure and taxa relative abundance [73••, 93]. This may be a result of hormonal interactions or of the offspring microbiota sex-driven effects. The latter is aligned with the finding that the infant baseline microbiota may determine the success of a maternal probiotic intervention aimed at preventing atopic dermatitis [83].

Notably, none of these studies used metagenomic approaches and thus it remains unknown if probiotics modulated microbial function. This is important because some pre-clinical studies found that microbial metabolites were altered in response to probiotics, including increased fecal short chain fatty acids in response to *Lacticaseibacillus rhamnosus* [62, 96] and decreased plasma trimethylamine N-oxide (TMAO) in response to *Lacticaseibacillus casei* [96]. Short-chain fatty acids such as acetate, propionate, and, in particular, butyrate are bacterial metabolites produced by fermentation of dietary fibers, and help maintain intestinal homeostasis [104]. TMAO is produced by liver oxidation of the intestinal microbial metabolite trimethylamine and is a marker of cardiovascular diseases [105]. It will be important for future studies to expand on these analyses to determine if programming effects of probiotics manifest at the functional level and to determine the quality and quantity of the microbial metabolites to which the host becomes exposed.

## Conclusions

**Fig. 1:**
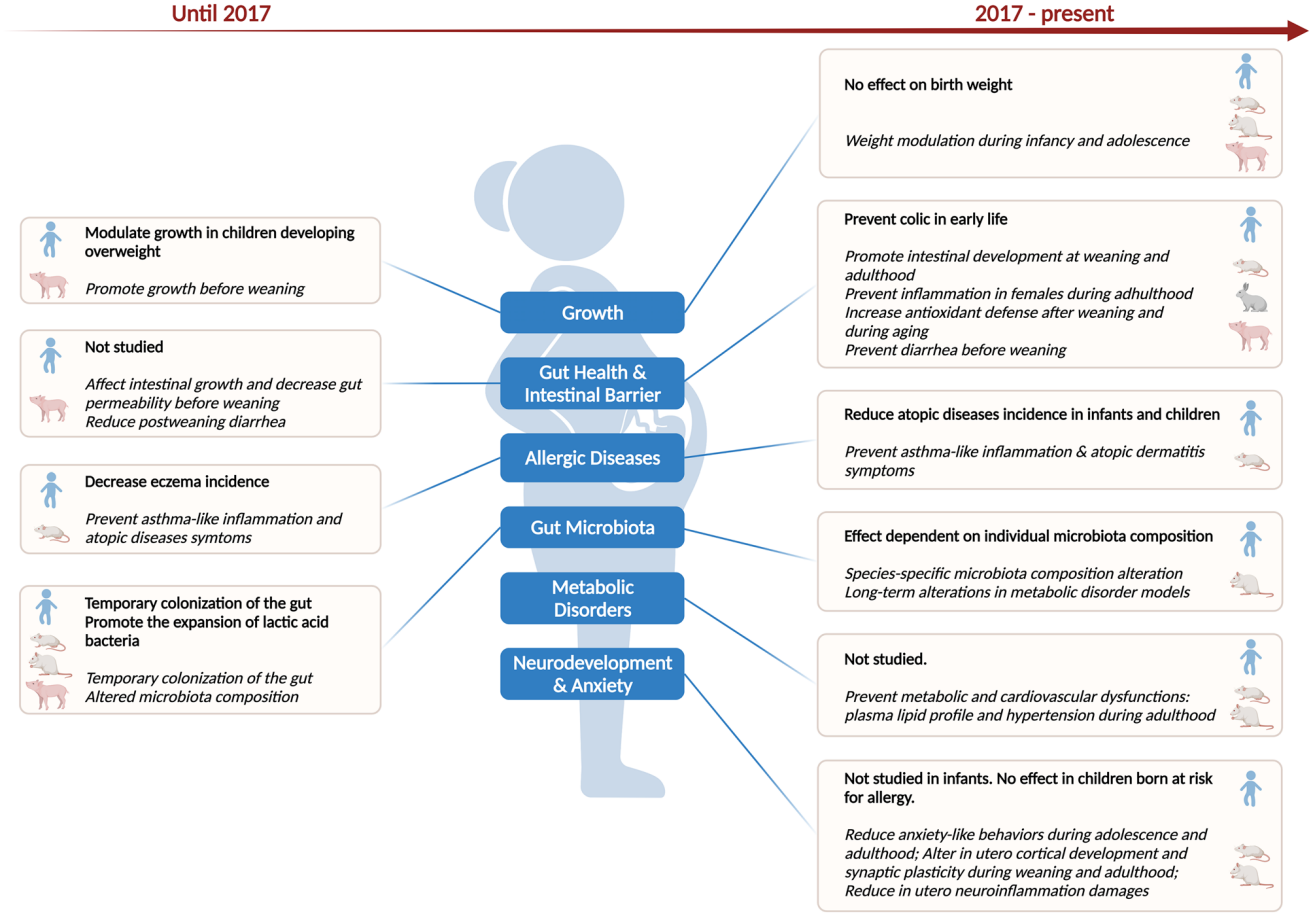
Offspring health outcomes programmed by maternal probiotic intake started prenatally. Previous knowledge (until 2017) included growth, gut health and intestinal barrier, allergic diseases, and gut microbiota findings. In addition to these outcomes, recent studies described effects on metabolic disorders, and neurodevelopment and anxiety. Clinical and pre-clinical findings are indicated in bold and italics, respectively. Icons depict findings assessed in infants or animal models (mice, rats, rabbits, or pigs). Created with BioRender.com

Here, we have reviewed recent pre-clinical and clinical studies investigating programming effects of maternally administered probiotics on offspring health. Studies published during the past 5 years expand on previous knowledge in the context of growth and gut health and additionally describe effects in the context of the metabolic syndrome and behavior (Fig. 1). Underlying mechanisms are also starting to be investigated. The study of probiotics in the context of DOHaD is in its infancy and it is not currently possible to make recommendations for clinical practice beyond allergic diseases. Pre-clinical studies are essential to investigate maternal programming effects of multiple outcomes in both male and female offspring in strictly controlled conditions and to elucidate molecular mechanisms. To encourage transparency and reproducibility of studies among research groups, it is important that these studies systematically report study details, including probiotic strain and dose along with timing and duration of probiotic exposure; the Animal Research: Reporting of *In Vivo* Experiments (ARRIVE) guidelines offer comprehensive recommendations for reporting [106].

While most clinical studies initiated the administration of probiotics towards the end of pregnancy and continued until 3 months of lactation, pre-clinical studies started at the beginning of pregnancy and continued until weaning. There are no studies that isolated the pregnancy period, and only one mouse and one clinical study began supplementation at pre-conception; it would be important to understand which are the windows of susceptibility for probiotics to positively affect the offspring. Offspring characteristics, including sex, can also play a role. The male sex is more susceptible to in utero programming [107] and different placental DNA methylation patterns have been observed for male and female infants [108]. Interestingly, four studies reviewed here proposed DNA methylation as an underlying mechanism for programming effects of probiotics [56•, 84, 100, 109•]. Effects could also be mediated by the microbiota, with preventative benefits in inflammation [73••]. Interestingly, a clinical study of probiotics administered during late pregnancy and lactation for the prevention of atopic dermatitis found that individual microbiota characteristics, in this case, representation of *Bifidobacterium dentium*, could determine the effects of the probiotic intervention [83]. It would be important to study the programming effects of probiotics in infants at risk for altered microbial maturation patterns, for example very low birth weight or malnourished infants. Microbial maturation continues throughout adolescence and studies have in fact found that probiotic effects might appear during puberty and adulthood [62, 87–89, 94–96]. Beyond allergy-related outcomes, current human studies report findings at birth or in infants up to 2 years of age; longer trials will provide a more comprehensive understanding of the programming potential of probiotics. Finally, the studies reviewed were performed in Northern Europe, Asia, and Oceania calling for more trials to be conducted in America and Africa and to encompass ethnically diverse populations.
